# A cohort study of the effectiveness of insecticide-treated bed nets to prevent malaria in an area of moderate pyrethroid resistance, Malawi

**DOI:** 10.1186/s12936-015-0554-1

**Published:** 2015-01-28

**Authors:** Kim A Lindblade, Dyson Mwandama, Themba Mzilahowa, Laura Steinhardt, John Gimnig, Monica Shah, Andy Bauleni, Jacklyn Wong, Ryan Wiegand, Paul Howell, John Zoya, John Chiphwanya, Don P Mathanga

**Affiliations:** Division of Parasitic Diseases and Malaria, US Centers for Disease Control and Prevention, 1600 Clifton Rd. NE MS A-06, Atlanta, GA 30333 USA; Malaria Alert Centre, Malawi College of Medicine, Blantyre, Malawi; National Malaria Control Programme, Ministry of Public Health, Lilongwe, Malawi

**Keywords:** Malaria, Insecticide-treated bed nets, Prevention, Vector control, Insecticide resistance

## Abstract

**Background:**

Insecticide-treated bed nets (ITNs) are the cornerstone of malaria control in sub-Saharan Africa but their effectiveness may be compromised by the spread of pyrethroid resistance among malaria vectors. The objective of this investigation was to assess the effectiveness of ITNs to prevent malaria in an area of Malawi with moderate pyrethroid resistance.

**Methods:**

One deltamethrin ITN was distributed in the study area for every two individuals in each household plus one extra ITN for households with an odd number of residents. A fixed cohort of 1,199 children aged six to 59 months was seen monthly for one year and at sick visits to measure malaria infection and use of ITNs. Insecticide resistance among malaria vectors was measured. The effect of ITN use on malaria incidence was assessed, adjusting for potential confounders using generalized estimating equations accounting for repeated measures.

**Results:**

There were 1,909 infections with *Plasmodium falciparum* over 905 person-years at risk (PYAR), resulting in an observed incidence of 2.1 infections per person-year (iPPY). ITNs were used during 97% of the PYAR. The main vector was *Anopheles funestus*: mortality in WHO tube assays after exposure to 0.05% deltamethrin was 38% (95% confidence interval (CI) 29–47), and resistance was due to elevated oxidase enzymes. After adjusting for potential confounders, the incidence of malaria infection among ITN users was 1.7 iPPY (95% CI 1.5-2.1) and among non-bed net users was 2.6 iPPY (95% CI 2.0-3.3). Use of ITNs reduced the incidence of malaria infection by 30% (rate ratio 0.7; 95% CI, 0.5-0.8) compared to no bed nets.

**Conclusion:**

ITNs significantly reduced the incidence of malaria infection in children in an area with moderate levels of pyrethroid resistance and considerable malaria transmission. This is the first study to show that ITNs provide protection in areas where pyrethroid-resistant *An. funestus* is the major malaria vector. Malaria control programmes should continue to distribute and promote ITNs in areas with low to moderate pyrethroid resistance; however, insecticide resistance may intensify further and it is not known whether ITNs will remain effective at higher levels of resistance. There is an urgent need to identify or develop new insecticides and technologies to limit the vulnerability of ITNs to insecticide resistance.

## Background

Bed nets have been used as a physical barrier to prevent nuisance mosquito biting since the Sixth Century BC, but were not used extensively for malaria control until after pyrethroid insecticides were applied to net material in the mid-1980s [1,2]. The combination of the insecticidal and irritant effect of the pyrethroids with the physical barrier of the bed net was found to reduce vector density, sporozoite rates, malaria parasite prevalence, disease incidence, and all-cause child mortality when evaluated both in clinical trials [3] and as part of routine public health programmes [4] in areas where the principal malaria vectors are largely endophagic (biting indoors) and endophilic (resting indoors). As a result, insecticide-treated bed nets (ITNs) are now the cornerstone of malaria prevention in Africa [5]. The World Health Organization (WHO) estimated that between 2010 and 2012, approximately 300 million ITNs were distributed in Africa, at a cost of more than US$1 billion for their purchase and distribution [6].

Pyrethroids are currently the only class of insecticide recommended for use on ITNs due to their low mammalian toxicity and long residual activity [7]. Concern over the potential impact of pyrethroid resistance on ITN effectiveness was expressed early in the development of ITNs for malaria control [8]. However, after manufacturing processes were developed to mass produce long-lasting ITNs, the potential risk from development of insecticide resistance was considered less important than protecting as many people as possible with a ‘brilliant’ new intervention [5]. As a result, household ITN ownership increased from less than 5% of sub-Saharan African households in 2000 to almost 60% in 2012 [6].

Pyrethroid resistance was first reported in *Anopheles gambiae s.s.* in West Africa in the early 1990s [9], and was later detected in *Anopheles funestus* and implicated in an epidemic of malaria in South Africa following the switch from DDT to pyrethroids for indoor residual spraying (IRS) [10]. Pyrethroid resistance in both vectors has since spread throughout the continent [11], at least partially as a result of widespread distribution of ITNs [12]. Pyrethroid resistance in anopheline mosquitoes may be due to target site resistance (point mutations that prevent insecticide from binding with receptor molecules on mosquito neurons); metabolic resistance (increased level of one or more enzymes capable of detoxifying or sequestering insecticides); or a combination of the two mechanisms [11,13]. Resistant mosquitoes are not killed or knocked down after contact with pyrethroid insecticides. As a result, although ITNs might continue to prevent blood feeding through the insecticide’s irritant properties and the bed net’s physical barrier, the community effect, which is dependent on the ability of ITNs to kill large numbers of adult mosquitoes, is likely to be compromised.

While there has been some limited evidence linking the operational failure of pyrethroid-based IRS to resistant vectors [14-16], there are as yet no epidemiologic data demonstrating that pyrethroid resistance reduces the effectiveness of ITNs to prevent malaria infection. Several studies of ITN efficacy or effectiveness conducted in pyrethroid-resistant areas have shown continued ability of ITNs to protect against malaria transmission when properly deployed [17-19]. A recent meta-analysis of entomologic outcomes from experimental hut trials conducted in areas with pyrethroid-resistant mosquitoes found that ITNs continued to reduce blood feeding and increase mosquito mortality compared to untreated bed nets, even in the areas with the highest levels of resistance [20]. However, the resistance profiling of the mosquitoes in these studies was inadequate, leaving significant uncertainties as to the resistance mechanisms responsible and thus limiting conclusions.

Malaria remains a significant problem in Malawi, where the entire population is at risk and it was estimated that 48% of the population resides in areas where the age standardized *Plasmodium falciparum* prevalence in children aged two to ten years is 40-50% [21] and more than 6.7 million clinical malaria cases occurred in 2010 [22]. The Malawi National Malaria Control Strategy 2011–2015 allocates almost one-third of its total five-year budget to prevention of malaria transmission through ITNs [23]. Since 2009, more than 20 million ITNs have been distributed in the country through mass campaigns as well as routine mechanisms such as antenatal and well-child clinics. As a result, household ownership of at least one ITN increased from 27% in 2004 to 55% in 2012, and use of ITNs by children less than five years old the night before the survey increased from <5% in 2000 to over 55% in 2012 [22].

WHO phenotypic resistance assays conducted in Malawi in 2007 found both major malaria vectors (*An. funestus* and *An. gambiae s.l.*) to be susceptible to all insecticides, including pyrethroids. Within three years, however, anopheline resistance to pyrethroids could be found in eight of 11 sites examined down the length of the country [24]. The frequency of resistance was higher in *An. funestus* than *An. gambiae s.l.*; biochemical assays demonstrated that the resistance mechanism was metabolically based for both species.

National malaria parasite prevalence in children less than five years in Malawi fell in 2012 to 28% from 43% in 2010 [25,26].However, prior to the encouraging 2012 results, several reports suggested that national scale-up of control activities had failed to substantially change the malaria burden in the country [21,27], and pyrethroid resistance was mentioned as a possible threat to successful malaria control [24]. Given the important role for ITNs in the Malawi national malaria control strategy, an evaluation was conducted of the effectiveness of ITNs in reducing malaria incidence among a cohort of children six to 59 months old in an area with moderate pyrethroid resistance.

## Methods

### Study area

**Figure 1:**
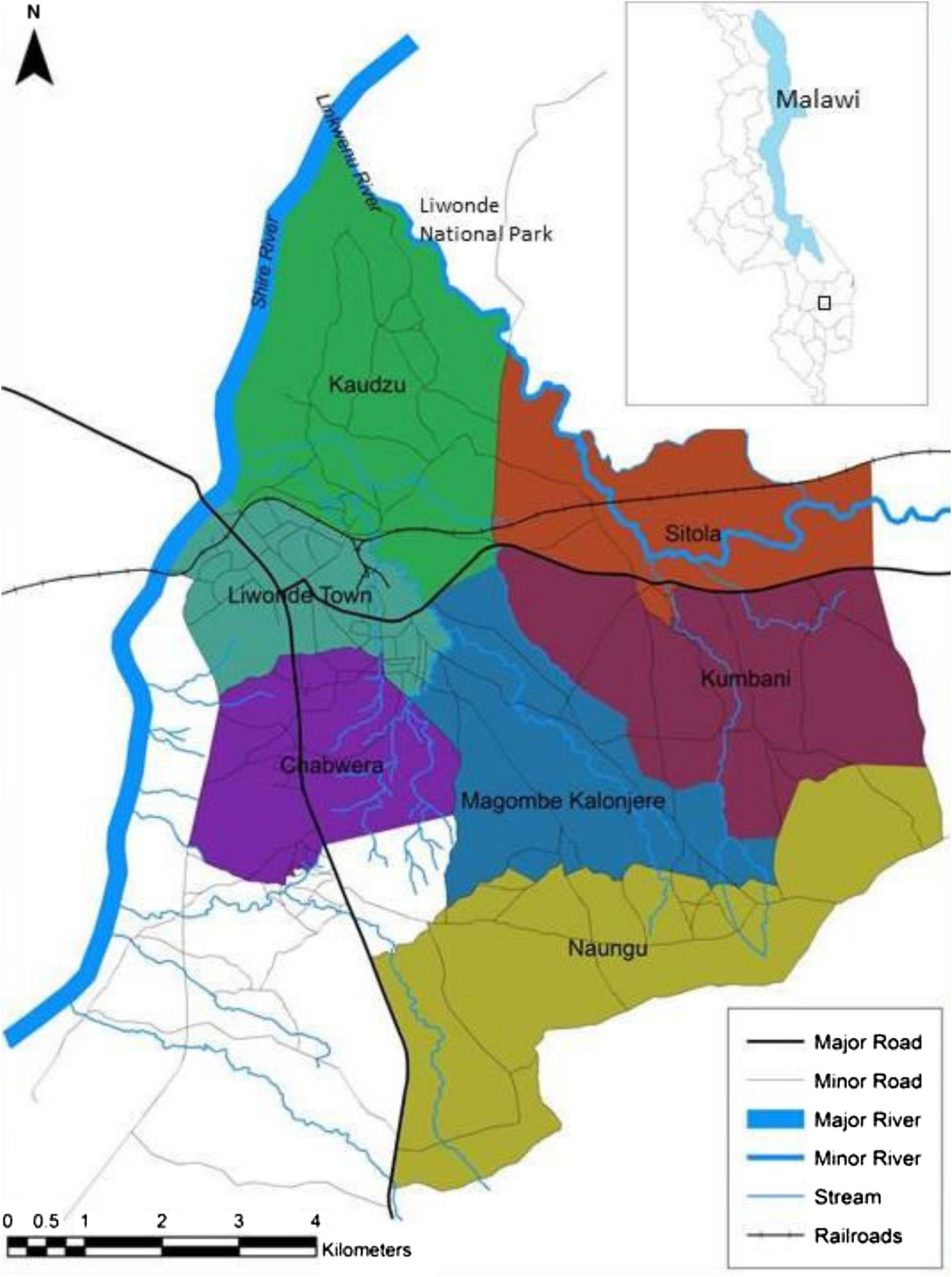
**Map of the study area, Liwonde, Malawi.**

The study was conducted in the Traditional Authority of Sitola in Machinga District, surrounding the town of Liwonde (500 m above sea level; −15°3'S 35°14'E). The study area is bordered on the west by the Shire River and on the northeast by the Likwenu River and Liwonde National Park (Figure 1). The population of the area is mostly of the Yao ethnic group, but both Chichewa and Chiyao are spoken. Annual rainfall is unimodal and approximately 1,000 mm falls between October and May, with daily temperature averaging 26°C in the rainy season and 21°C in the dry season. In this area, malaria transmission is intense and perennial, peaking during the rainy season. Entomological monitoring in Machinga District by the Malaria Alert Centre of the Malawi College of Medicine (Blantyre, Malawi) prior to the start of the study found a moderate level of pyrethroid resistance of *An. funestus* to 0.05% deltamethrin (38% mortality, 95% confidence interval (CI) 28–49) (TM, pers comm). This area has never received indoor residual spraying for malaria control.

### Study design and population

The incidence of malaria infection was compared between users and non-users of ITNs in a fixed cohort of children aged six to 59 months who were followed for 12 months after they were cleared of infection with artemether-lumefantrine. Preliminary household mapping was conducted in late 2011 and the six villages closest to Liwonde town, but excluding the urban area itself, were selected for inclusion. Households in these villages were geocoded in February and March 2012 and all children six to 59 months old were invited to participate in the survey, with some households contributing more than one eligible child. Incident cases of malaria infection were identified through a combination of active (routine monthly visits) and passive (sick visits) surveillance.

### ITN distribution

The initial household census was used to determine the number of ITNs needed per house by allocating one ITN per two household residents plus one extra for households with odd numbers of residents. The long-lasting bed nets distributed were Permanet 2.0 (Vestergaard, Lausanne, Switzerland), treated with deltamethrin, and a total of 5,146 ITNs were distributed to household heads during the month of May 2012. The allocation algorithm used in the area was the same as that used by the National Malaria Control Programme in the national ITN distribution that took place in July 2012 (JZ, pers comm).

### Sample size determination

The sample size was based on a generalized estimating equations modelling approach with a Poisson distribution and accounted for the correlated nature of repeated measurements on study children using an exchangeable correlation structure [28]. The incidence among children who did not use ITNs (projected to be 30% of the population after the ITN distribution) was estimated to be 1.0 episodes per child-year, and the power to detect a 30% reduction in incidence among those using ITNs (projected to be 70% of the population after ITN distribution) was set at 70%, with the probability of committing a Type 1 error equal to 5%. The resulting calculation yielded 684 ITN users and 297 non-ITN users for a total of 981 children. Assuming a 5% refusal rate, 15% loss-to-follow-up over 12 months, and a mortality rate of 1.5% per year resulted in a total of 1,335 children to be enrolled at baseline. Under the assumption that in the study area, 60% of households had at least one child aged six to 59 months, a total of 2,225 households would be needed. As 2,300 households were mapped in the study area, all age-eligible children were invited to participate in the cohort study.

### Enrolment and monthly visits

Children were eligible to participate if they were between six and 59 months as of 1 March, 2012, not taking daily cotrimoxazole for HIV infection or exposure, weighed 5 kg or more and planned to stay in the study area for at least one month.

At enrolment and all monthly visits, children and their caregivers were invited to a central location near their home; no reimbursement or incentive was provided to caregivers to attend the routine monthly visits. At enrolment and the tenth monthly visit, children had their height and weight measured. At each monthly visit, axillary temperature was measured and a finger stick was performed to collect a blood sample for thick and thin blood smears and haemoglobin (Hb) measurement using a Hemocue® (Angelholm, Sweden) portable haemoglobinometer. At enrolment, a dried blood spot was also made for *Plasmodium* testing by nested polymerase chain reaction (PCR) at the Malawi College of Medicine in Blantyre [29]. All samples collected at enrolment were tested for the presence of *P. falciparum*, *Plasmodium malariae, Plasmodium ovale,* and *Plasmodium vivax*. At monthly visits, malaria testing was conducted using a histidine-rich protein 2 (HRP-2) *P. falciparum* malaria rapid diagnostic test (RDT; SD Bioline Malaria Ag Pf® ref. 05FK53, Kyonggi-do, Republic of Korea).

At enrolment and all monthly visits, parents or caregivers were questioned regarding the child’s two-week illness history, household bed net ownership and bed net use. Bed net use by the child was referenced for the night before the survey, the two-week period before the survey and for typical use during the current season. Study ITNs were not distributed until after enrolment in May 2012, and many residents retained their older bed nets. Therefore, the age of the bed net used by the child was requested along with the caregiver’s assessment of the condition (presence/absence of holes, the number of holes larger than a fist and the number larger than a head).

At enrolment, all children were provided with a full, weight-appropriate treatment course of dispersible artemether-lumefantrine (Coartem®-D, Novartis, Basel, Switzerland); the first dose was observed by study staff, and parents or caregivers were instructed on how and when to give the remaining doses. At subsequent visits, only children with positive RDT results were treated with an appropriate anti-malarial.

Study staff attempted to contact any child who did not attend the monthly visit to schedule a make-up visit. Any child who did not attend three consecutive visits for any reason was withdrawn from the study and censored as of their last visit date.

### Sick visits

During the study period, caregivers were encouraged to bring participants to the study clinic located at Machinga District Hospital, the only health care facility within 30 km of Liwonde Town, if they became sick. Artemisinin-based combination therapy is free at public health facilities, and 91% of children under five years of age with fever in the two weeks before the Malawi Malaria Indicator Survey in 2012 and treated with an anti-malarial received an artemisinin-based combination therapy, suggesting limited use of private physicians and drug shops [26]. Sick children were examined by a study clinician who measured axillary temperature and took a finger-prick blood sample for malaria testing using a RDT, and Hb testing with a Hemocue®. The same questionnaire used at enrolment and the monthly visits was employed to elicit a two-week period illness history and bed net use from the child’s caregiver. Caregivers were reimbursed for transport to the study clinic for one sick visit per month.

### Confirmation of ITN use

The reliability of caregiver-reported ITN use was evaluated twice during the study period (JW*,* pers comm). Briefly, children attending monthly visits between December 2012 and January 2013 and September and October 2013 were randomly selected for follow-up home visits, which occurred between zero and eight days after the monthly visit. Caregiver responses to questions about ITN use at the monthly visits were compared to the same questions asked at the home visit plus visual inspection of the ITN reported to be used by the child. The proportion of positive and negative agreement of ITN use between caregiver report and home visit were calculated and 95% CI were constructed using bootstrapping.

### Entomologic surveillance and resistance monitoring

The study area was divided into ten clusters and every two weeks pyrethroid spray catches (PSCs) were conducted in ten houses per cluster using standard methods [30]. Sexing and morphologic species identification was conducted in the laboratory at the Malaria Alert Centre. *Anopheles funestus* and *An. gambiae s.l.* mosquitoes were tested for the presence of sporozoites in their salivary glands using standard procedures [31]. Phenotypic pyrethroid resistance was evaluated in the study area during the course of the study using the standard WHO tube assay with two to five days old *An. funestus* and *An. gambiae s.l.* reared from eggs or larvae [32].

To determine whether the mechanism of resistance was an elevated oxidase or esterase enzyme, mosquitoes were pre-exposed to the synergist, piperonyl butoxide (PBO), for one hour before the WHO tube assay was conducted. Synergists are non-toxic to insects but improve the effect of an insecticide by inhibiting the metabolic enzymes (oxidases and esterases) that cause mosquitoes to be resistant. Mosquitoes were exposed to PBO in 250 ml Wheaton bottles. The bottles were coated by adding 1 ml of a stock solution of 400 mg/L of PBO (corresponding to a dose of 400 ml/bottle) in acetone, rotating the bottles to ensure all surfaces were covered and then allowing the acetone to evaporate. Two to five days old female mosquitoes were added to the bottle and exposed for one hour. The mosquitoes were then removed and exposed to 0.05% deltamethrin or 0.75% permethrin in WHO tube assays as described above [33].

Assays were performed to detect mutations in the knockdown resistance (*kdr*) locus using either a leg or wing removed from the mosquito corpse of mosquitoes for which species identification was confirmed molecularly. Stratified samples of mosquitoes of both species determined by the WHO resistance assay to be resistant or susceptible to permethrin or deltamethrin were randomly selected for testing for commonly reported *kdr* mutations. Two different assays were utilized depending on the species being tested: Huynh *et al*. for *An. gambiae s.l.* and Morgan *et al*. for *An. funestus* [34,35]. All assays were performed without modification and PCR amplicons were visualized on a 1.5% 0.5X tris-borate-EDTA agarose gel stained with ethidium bromide and visualized in a Gel Doc-It^2^ UV imager (UVP LLC, Upland, CA, USA). Further sequencing was done on *An. funestus* samples to determine if single nucleotide polymorphisms associated with resistant populations were present within the amplified region. Samples were processed using BigDye® Terminator v3.1 (Life Technologies, Grand Island, NY, USA), purified with BigDye® XTerminator and sequenced on an ABI 3500 sequencer (Applied Biosystems, Foster City, CA, USA). Sequences were aligned using Lasergene® SeqMan (DNASTAR, Madison, WI, USA) and confirmed using the basic local alignment search tool feature.

The WHO definition considers insecticide resistance to be confirmed when mortality of mosquitoes exposed to insecticides in bioassays is <90% [32]. The level of resistance was classified according to Strode *et al*. [20].

Mosquitoes of both species (*An. funestus* and *An. gambiae s.l.*) were selected from the mosquitoes alive or killed by the resistance bioassay and tested using PCR to determine the sibling species. Mosquitoes of the *An. gambiae* complex were identified using the methods described by Wilkins *et al*. [36] while members of the *An. funestus* group were identified using a modification of the protocol described by Koekemoer *et al*. with intentional mismatched primers [37,38]. For *An. funestus*, specimens were tested with primers for *An. funestus s.s.*, *Anopheles vaneedeni*, *Anopheles rivulorum*, *An. rivulorum*-like, *Anopheles parensis,* and *Anopheles leesoni. Anopheles gambiae s.l.* were tested with primers for *An. gambiae s.s*., *Anopheles arabiensis*, *Anopheles coluzzii*, *Anopheles quadriannulatus* (species A) and *Anopheles melas/merus*. Mosquitoes that did not amplify against one of these primers were retested with universal primers for the internal transcribed spacer 2 region to determine whether the lack of amplification was due to poor specimen preservation.

### Data management and analysis

The main outcome was *P. falciparum* parasitaemia at a sick or monthly visit as measured by RDT. The main exposure variable was bed net use the night before the visit, categorized as ITN, untreated bed net (UTN) or no bed net. Bed nets used between enrolment and the end of the ITN distribution were classified as a UTN if they were reported to be 36 months or older; however, as of 1 June, 2012, after ITNs were distributed by the project, no bed nets were considered to be untreated. Children were described as anaemic (Hb <11 g/dl) or not anaemic (Hb ≥11 g/dl).

A number of potential confounders was measured and included in the analysis. Baseline parasitaemia was measured by PCR and used to indicate previous exposure to malaria infection. A proxy measure of individual exposure to malaria transmission was generated by using the inverse distance-weighted (IDW) malaria prevalence in children within 1 km, divided into terciles [39]. Household altitude was divided into terciles. Household ownership of key assets was analysed using principal component analysis and the coefficient of the first component was used to generate a household asset score [40]. Assets included electricity, paraffin lamp, battery lamp radio, television, cell phone, mattress, sofa set, table and chairs, refrigerator, bicycle, motorcycle, car, source of water, type of toilet, type of floor material, type of roof material, type of wall material and number of sleeping rooms. Children were divided into terciles according to their household’s asset score. Stunting (height-for-age) and wasting (weight-for-age) were defined as Z-scores < −2 compared to the WHO growth reference charts by age and sex [41]. Children were remeasured in the tenth month of the survey and their nutritional status was allowed to change. Because of the potential for the density of ITNs around a participant to create a mass effect, participants were divided into terciles based on the total number of ITNs found in households within 300 m; this radius was based on data from a cluster-randomized trial of ITNs in western Kenya that found a mass effect within 300 m [42]. Data on household ITN ownership from the household mapping exercise before the start of the study were used to calculate ITN density for participants between enrolment and the time of the ITN distribution. After the ITN distribution was completed, a new density calculation was made assuming the number of ITNs per household matched the distribution algorithm of one ITN per every two household residents with one extra for households with an odd number of residents. The seasonality of malaria transmission was accounted for by dummy variables indicating visits during the high (April-June 2012 and January-March 2013) or low (July-December 2012) malaria transmission season.

Data from monthly and sick visits were combined into one dataset and time between visits was calculated as person-time at risk. After every positive result treated with artemether-lumefantrine, including the baseline treatment dose, 10.5 days were subtracted from the person-time at risk to account for the half-life of lumefantrine [43].

As febrile children who came for sick visits in between monthly visits would have been tested again for malaria with an RDT, there was the potential that the second of sequentially positive RDTs was a false positive due to persistent antigen from a recently treated infection. Follow-up of infected children after malaria treatment in Uganda found that the median duration of persistent antigenaemia using HRP2-based RDTs was 35 days (95% CI 33–37), although the possibility of re-infection could not be ruled out [44]. A mathematical model of HRP2 antigenaemia found that persisting antigen caused tests to remain positive for up to seven days after treatment, depending on the length and density of infection before treatment [45]. In this study, there were 972 pairs of sequential visits (out of a total of 13,166 visits) in children with positive RDT results. The median time between these paired, positive sequential visits was 17 days. To attempt to minimize the potential that the second of these two visits was a false positive, second visits > =17 days were considered new infections, whereas second visits with a positive RDT that occurred <17 days after an earlier positive visit were considered false positives. Recognizing that there is no clear cut-off to determine whether a second positive RDT was due to a new infection or persistent antigenaemia, a sensitivity analysis was conducted by varying the cut-off +/−one week in either direction, i.e., cut-offs of 10 and 24 days.

SAS® (v. 9.3, SAS Institute, Inc., Cary, NC, USA) was used for all analyses. Data were analysed using Poisson regression with a generalized estimating equations approach (PROC GENMOD) to account for the extra correlation from repeated measures on the same child [46]. Both the exposure variable and covariates were allowed to be time varying. Log-transformed person-time was used as an offset and an exchangeable working correlation structure was specified. Any covariate with a p-value of >0.1 in univariate analysis was included in the multivariate model. Rate ratios (RR) and 95% CI were calculated from model parameters and model-adjusted incidence rates for covariates are presented. Protective effectiveness (PE) was calculated as 100%*(1-R_1_/R_0_) where R_1_ is the rate among ITN or UTN users and R_0_ is the rate among non-bed net users. The attributable rate difference was calculated as R_1_-R_0_ and interpreted as the number of malaria infections prevented by ITNs annually.

### Human subjects

Household heads provided verbal consent to have their household mapped and residents enumerated. Parents or caregivers provided verbal consent to have their child screened for eligibility, and read and signed written informed consent forms if the child was found to be eligible. Households selected for PSCs provided verbal consent. The protocol was reviewed and approved by the Malawi College of Medicine Research Ethics Committee (Blantyre, Malawi) and by the US Centers for Disease Control and Prevention institutional review board (Atlanta, GA, USA).

## Results

### Study profile and baseline characteristics

**Figure 2:**
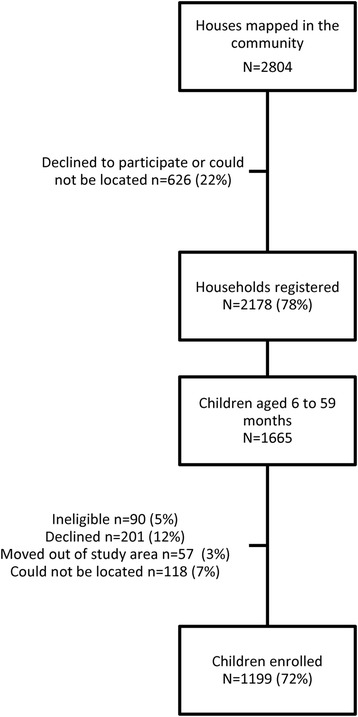
**Study profile, Liwonde, Malawi 2012–2013.**

There were 2,804 houses in the study area, and 2,178 (78%) agreed to be mapped and enumerated (Figure 2). A total of 1,665 children between six and 59 months old were registered and 1,199 (72%) enrolled from 907 households. There were 268 (29%) households with more than one child enrolled. A total of 175 (10%) children could not be located during the enrolment period or were found to have moved out of the study area; 201 (12%) children declined to participate, and 90 (5%) were found to be ineligible. Comparing the enrolled children with those who declined to participate or could not be located, males (78%) were more likely to be enrolled than females (74%; Chi square test p = 0.04), but those enrolled had a similar median age (30 months) to those not enrolled (29 months; Wilcoxon signed-rank test p = 0.80). There was no significant difference in enrolment between children who reported sleeping under a bed net (76%) and those who did not (73%; Chi square test p = 0.26).

Median follow-up time of the cohort of 1,199 children was 0.96 years (interquartile range (IQR) 0.92, 0.97) and 1,018 (85%) children completed one year of follow-up (Table 1). Reasons for early exit from the cohort included: consent withdrawn or child moved out of the study area (n = 145, 12%), started cotrimoxazole therapy (n = 3, 0.2%), died (n = 2, 0.2%), and could not be traced (n = 31, 3%). The participants had 13,166 encounters with the study and the time at risk for the whole cohort was 905 person-years (after removing non-risk periods due to anti-malarial treatment). Attendance at monthly visits varied between 90% in May 2012 and 74% in December 2012; at least one sick visit was made by 67% (n = 803) of the participants, and the median number of sick visits among them was 2 (IQR 1, 4).

**Table 1:** **Descriptive characteristics of the study cohort, Liwonde, Malawi 2012-2013**

**Characteristic**	**Results**
Number of children enrolled	1,199
Number of households included	907
Median age at enrolment in months (IQR)*	30 (17, 44)
Number of children who completed one year follow-up, n (%)	1,018 (85)
Person-years at risk	905
Female, n (%)	579 (48)
*Plasmodium* infection at baseline (PCR), n/N (%)*	440/1,192 (37)
Median IDW malaria prevalence <1 km (IQR)*	35% (24, 48)
Anaemic (Hb <11 g/dl), n (%)	860 (72)
Caregiver completed primary school, n (%)	215 (18)
Bed net used, n/N (%)	922/1,175 (78)
ITN used, n/N (%)	516/1,174 (44)
ITN has ≥1 hole fist-sized or larger, n/N (%)	110/404 (21)
Median number of ITNs within 300 m (IQR)	
Baseline	8 (5, 21)
After ITN distribution	48 (27, 116)
Malaria infections**	1,909
Median malaria incidence per person-year (IQR)	1.2 (0, 3.6)

*IQR = Interquartile range; PCR = polymerase chain reaction; IDW = inverse distance-weighted; Hb = haemoglobin; PPY = per person year.

**As measured by malaria rapid diagnostic tests.

Slightly less than half (48%) of the enrolled children were female (Table 1). At baseline, 37% of children were infected with malaria as confirmed by PCR, the majority (97%) with *P. falciparum,* and the remainder with *P. malariae* and *P. ovale*. The median IDW prevalence of malaria in children within 1 km was 35% (IQR 24, 48). The median Hb measurement was 10.0 g/dl (IQR 8.7, 11.1) and 72% of children were anaemic. A third of children (32%) were stunted and 3% met the definition of wasted. Less than one-fifth (18%) of the caregivers had completed primary school.

At baseline, bed net use the night before the survey was reported for 78% of children, but only 44% were reported to have used an ITN. Use of an ITN the night before the survey was strongly associated with use of an ITN over the previous fortnight: 97% of those who used an ITN the night before the survey had used an ITN every night out of the previous 14 nights and the remainder had used an ITN at least once in the last two weeks. Among those reporting not using an ITN the night before the survey, 99% reported not using an ITN at all during the previous 14 nights. A large proportion (59%) of the ITNs were reported to have holes, with one-fifth (21%) reported to have at least one hole fist-sized or greater. The median number of ITNs within 300 m of participants at baseline was 8 (IQR 5, 21).

### Malaria incidence

**Figure 3:**
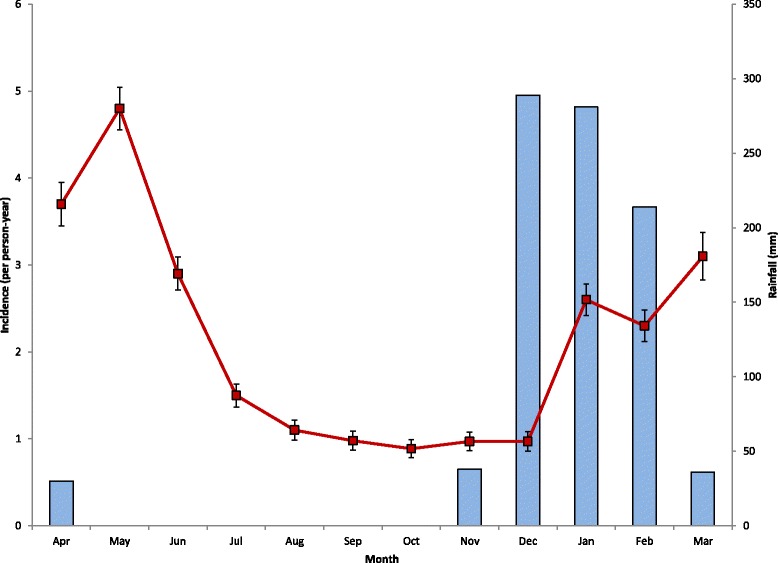
**Monthly incidence of malaria infection in a fixed cohort of children age six to 59 months at baseline and monthly rainfall, Liwonde, Malawi 2012–2013.** Error bars indicate the standard error of the rate.

Between April 2012 and March 2013 there were 1,909 infections with *P. falciparum* identified, most (65%) through active surveillance at monthly visits (Table 1). The median incidence per child was 1.2 infections per person-year (iPPY) (IQR 0, 3.6), and the overall incidence was 2.1 iPPY. A third of the children (32%) never had documented malaria infection during the study, whereas 44% had two or more infections. During the study period, which crossed two rainy seasons, there were two peaks of transmission corresponding to the end of the 2012 rainy season (May 2012) and the last month of the 2013 rainy season included in the study survey (March 2013; Figure 3).

### Use of ITNs over time

Information on child’s ITN use was recorded for 895 person-years at risk (PYAR), and ITN use the night before the survey was reported for 864 (97%) PYAR, UTN use for 17 (2%) PYAR and no bed net for 15 (2%) PYAR. To measure the reliability of caregiver-reported ITN use at the routine monthly surveys, study staff visited 211 randomly selected children from December 2012-January 2013 and 325 children between September and October 2013 (JW, pers comm). The observed proportions of positive agreement between caregiver report and home visit on ITN use by the child in the first and second surveys were 98.8% (95% CI 97.6-99.8) and 93.3% (95% CI 91.2-95.3), respectively. The proportions of negative agreement in the first and second survey were 28.6% (95% CI 0–75) and 20.0% (95% CI 0.1-35.0).

### Entomologic surveillance and resistance monitoring

**Figure 4:**
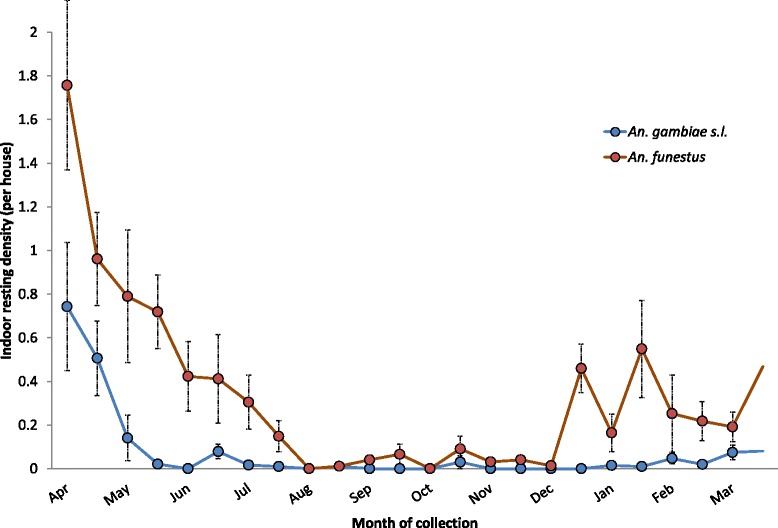
**Indoor resting density of malaria vectors in Liwonde, Malawi, 2012–2013.** Error bars indicate the standard error of the mean, accounting for the sampling design.

There were 885 female anophelines collected through PSC; 743 (84%) were identified as *An. funestus* Giles and 142 (16%) as *An. gambiae s.l*. Mean indoor resting density for both species was highest in April 2012 and again between December 2012 and March 2013 (Figure 4). A total of 711 of the *An. funestus* and 114 of the *An. gambiae s.l.* collected by PSCs were tested for sporozoites and 30 (4%) and two (2%) were positive, respectively.

Results of the phenotypic resistance assays are shown in Table 2. Mortality of *An. funestus* after exposure to 0.05% deltamethrin and 0.75% permethrin was 38 and 25%, respectively. After exposure to the synergist (PBO), mortality rates with these insecticides rose to 89 and 98%, respectively. Mortality of *An. gambiae s.l.* to 0.05% deltamethrin and 0.75% permethrin was 53 and 65%, and exposure to PBO increased mortality from 0.75% permethrin to 100%. No testing of *An. gambiae s.l.* to 0.05% deltamethrin after PBO exposure was conducted due to low numbers of mosquitoes. Taken together, the results of increased pyrethroid susceptibility after exposure to PBO implicate elevated oxidase enzymes as the primary resistance mechanism.

**Table 2:** **Results of resistance testing using the WHO tube assay with two to five days old mosquitoes with and without pre-exposure to a synergist, Liwonde, Malawi 2012-2013**

		**Without pre-exposure to PBO** ^**a**^	**With pre-exposure to PBO**
**Mosquito species**	**Insecticide**	**% mortality (95% CI)** ^**b**^	**% mortality (95% CI)**
		**(No. died/tested)**	**(No. died/tested)**
*An. funestus*	Deltamethrin 0.05%	38 (29–47) (58/152)	89 (37–100) (34/38)
	Permethrin 0.75%	25 (8–42) (23/93)	98 (93–100) (89/91)
*An. gambiae s.l.*	Deltamethrin 0.05%	53 (24–81) (49/93)	
	Permethrin 0.75%	57 (32–82) (65/114)	100 (90–100) (27/27)

^a^ Mosquitoes with and without pre-exposure to PBO for one hour before resistance testing using the WHO tube assay.

^b^ 95% confidence intervals calculated taking clustering by tube into account.

Of the 22 *An. gambiae s.l.* tested, none possessed either of the two *kdr*-like mutations L1014F (West African *kdr*) or L1014S (East African *kdr*) reported in *An. gambiae s.s*. Of the 20 *An. funestus* samples chosen for additional sequencing, seven resistant and eight susceptible samples yielded complete sequences. Based on alignments with the *An. funestus* FUMOZ laboratory colony, which originates from Mozambique and does not possess *kdr*, none had any single nucleotide polymorphisms that were unique to either the resistant or susceptible populations. In summary, neither vector species showed evidence for the presence of *kdr* genes.

There were 23 *An. funestus s.l.* identified to species out of 100 that survived exposure to pyrethroids, and 12 tested out of 39 that were killed by exposure to pyrethroid and all (100%) were found to be *An. funestus s.s*. There were 17 *An. gambiae s.l.* tested that survived the pyrethroid exposure and 22 that did not, and 16 and 22, respectively, were identified to species; 100% of the surviving mosquitoes and 95% (21/22) of the killed mosquitoes were found to be *An. arabiensis*. One (5%) of the killed mosquitoes was identified as *An. gambiae s.s*.

### Bed nets and malaria incidence

In univariate analysis, children between six and 11 months old experienced twice the malaria incidence than those older than 12 months; malaria infection at baseline was also associated with almost twice the malaria incidence of those without infection at baseline (Table 3). Use of ITNs, but not UTNs, was associated with a 60% reduction in the incidence of malaria compared to children who did not use bed nets. Poorer children and those whose caregivers had not completed primary school had higher rates of malaria. Children living at the lowest altitudes had a 40-60% increase in malaria incidence compared to those at the highest elevations. Children living in households with the lowest numbers of ITNs within 300 m experienced almost twice the incidence of malaria than children living with the highest ITN density, whereas the children living with the lowest IDW malaria prevalence within 1 km experienced 20-40% reductions in malaria incidence. Malaria incidence was highest after the rains from April to June 2012, with an incidence of 4.1 iPPY, and lowest during the dry season from July to December 2012, with an incidence of 0.9 iPPY.

**Table 3:** **Predictors of malaria incidence in a fixed cohort of 1,199 children age six to 59 months at baseline, Liwonde, Malawi 2012- 2013** ^**a**^

				**Univariate**			**Multivariate**		
**Characteristic**		**Malaria infections (n)**	**Person-years at risk**	**Observed incidence PPY**	**Rate ratio (95% CI)**	**P value**	**Adjusted incidence** ^**b**^ **PPY (95% CI)**	**Rate ratio (95% CI)**	**P value**
Age (months)	6-11	64	17	3.7 (2.7-5.0)	2.2 (1.6-3.0)	<0.0001	2.1 (1.5-2.9)	1.0 (0.8-1.4)	0.84
	12+	1845	891	1.7 (1.6-1.8)	1.0		2.0 (1.8-2.3)	1.0	
Sex	Female	905	433	1.7 (1.6-1.9)	1.0 (0.9-1.1)	0.96			
	Male	1004	471	1.7 (1.6-1.9)	1.0				
Baseline *Plasmodium* infection	Positive	948	329	2.4 (2.2-2.7)	1.7 (1.5-2.0)	<0.0001	2.7 (2.2-3.3)	1.7 (1.5-1.9)	<0.0001
	Negative	960	573	1.4 (1.3-1.5)	1.0		1.6 (1.3-2.0)	1.0	
Bed net use	ITN	1710	864	1.6 (1.5-1.7)	0.4 (0.3-0.5)	<0.0001	1.7 (1.5-2.1)	0.7 (0.5-0.8)	<0.0001
	UTN	92	17	4.9 (4.1-5.9)	1.1 (0.8-1.5)	0.53	2.0 (1.6-2.6)	0.8 (0.6-1.0)	0.06
	No bed net	86	15	4.4 (3.5-5.7)	1.0		2.6 (2.0-3.3)	1.0	
Wealth index	Poorest	725	290	2.1 (1.9-2.3)	1.5 (1.3-1.8)	<0.0001	2.3 (1.9-2.8)	1.3 (1.1-1.5)	0.0008
	Middle	647	296	1.8 (1.6-2.0)	1.3 (1.1-1.6)	0.001	2.2 (1.8-2.7)	1.2 (1.1-1.5)	0.01
	Least poor	495	309	1.4 (1.2-1.6)	1.0		1.8 (1.4-2.2)	1.0	
Caregiver completed primary	Yes	239	164	1.2 (0.9-1.4)	0.6 (0.5-0.8)	<0.0001	1.9 (1.5-2.4)	0.8 (0.7-1.0)	0.02
	No	1628	730	1.9 (1.7-2.0)	1.0		2.3 (1.9-2.8)	1.0	
Altitude	Lowest	680	300	1.9 (1.7-2.1)	1.4 (1.2-1.7)	0.0001	2.4 (1.9-2.9)	1.5 (1.3-1.8)	<0.0001
	Middle	727	294	2.1 (1.9-2.4)	1.6 (1.3-1.9)	<0.0001	2.4 (2.0-2.9)	1.5 (1.3-1.8)	<0.0001
	Highest	497	307	1.3 (1.2-1.5)	1.0		1. (1.3-2.0)	1.0	
Number of ITNs <300 m	Lowest	586	249	2.0 (1.8-2.3)	1.7 (1.4-2.1)	<0.0001	2.1 (1.7-2.6)	1.1 (0.9-1.4)	0.39
	Middle	737	313	2.1 (1.9-2.3)	1.7 (1.5-2.1)	<0.0001	2.2 (1.8-2.7)	1.2 (0.9-1.4)	0.17
	Highest	389	272	1.2 (1.0-1.4)	1.0		1.9 (1.5-2.4)	1.0	
IDW malaria prevalence	Lowest	474	294	1.3 (1.1-1.5)	0.6 (0.5-0.7)	<0.0001	1.7 (1.3-2.1)	0.7 (0.5-0.8)	0.0002
	Middle	610	304	1.7 (1.5-1.9)	0.8 (0.6-0.9)	0.0003	2.2 (1.7-2.7)	0.9 (0.7-1.0)	0.04
	Highest	820	302	2.3 (2.1-2.5)	1.0		2.5 (2.1-3.1)	1.0	
Season	Apr-June	844	178	4.1 (3.8-4.4)	2.3 (2.0-2.7)	<0.0001	4.6 (3.9-5.5)	2.3 (2.0-2.6)	<0.0001
	July-Dec	556	486	0.9 (0.8-1.0)	0.5 (0.4-0.6)	<0.0001	1.0 (0.8-1.2)	0.5 (0.4-0.6)	<0.0001
	Jan-Mar	509	241	1.8 (1.6-2.0)	1.0		2.0 (1.6-2.5)	1.0	

^a^ PPY = per person year; IDW = inverse distance-weighted; ITN = insecticide-treated bed net; UTN = untreated bed net; CI = confidence interval.

^b^ Incidence is model-adjusted for all other variables in the column.

After controlling for all variables significantly associated with malaria incidence in univariate analyses, use of an ITN was associated with a protective effectiveness of 30% (RR 0.7, 95% CI 0.5-0.8; Table 3). UTNs were not statistically associated with malaria incidence (RR 0.8, 95% CI 0.6-1.0). Results were not sensitive to the time period used to define whether the second of two sequential visits with positive RDT results was considered a new infection: with a cut-off of 10 days, use of an ITN was associated with a 30% reduction in malaria incidence (RR 0.7, 95% CI 0.5-0.9), whereas a cut-off of 24 days resulted in a 40% reduction (RR 0.6, 95% CI 0.5-0.7).

The model-adjusted incidence of malaria among ITN users was 1.7 iPPY (95% CI 1.5-2.1) and among UTN users was 2.0 iPPY (95% CI 1.6-2.6), whereas among children who did not use any bed net, the model-adjusted incidence was 2.6 iPPY (95% CI 2.0-3.3). The attributable rate difference for ITNs (subtracting the incidence rate among those who used ITNs from the incidence rate of those who did not, i.e. 2.6-1.7 iPPY) was 0.9 malaria infections averted per person-year, or 815 malaria infections averted in the study population. Therefore, for every ten children protected by ITNs, nine malaria infections were prevented annually.

## Discussion

ITNs were found to reduce the incidence of malaria by 30% among children six to 59 months old in an area of Malawi where deltamethrin killed only 38% of *An. funestus,* the main malaria vector, and 53% of *An. gambiae s.l*., the secondary vector, in WHO tube assays. Prior to the continent-wide increase in pyrethroid resistance, randomized controlled trials of the efficacy of ITNs compared to no bed nets in areas with similar malaria incidence to Malawi showed ≥50% reductions in the incidence of malaria [47-49]. It is possible, therefore, that in the absence of pyrethroid resistance, ITN effectiveness might have been higher in this population. However, the lower effectiveness of ITNs in this study compared to earlier trials also could be due to factors related to the study design (e.g., observational cohort *vs* randomized controlled trial) and analytical methods. Additionally, the high rate of ITN use in this population may have suppressed transmission, benefitting non-bed net users and reducing differences between users and non-users of ITNs.

ITNs prevent malaria through several modes of action: the insecticide deters mosquitoes from entering houses, irritates the mosquitoes that encounter the insecticide, causing them to leave the area prematurely, and kills the mosquitoes that acquire a lethal dose. In addition, ITNs provide a physical barrier to blood feeding. This latter mode of action is the only one that does not depend on the insecticide, and potentially could account for continued ITN effectiveness in areas with pyrethroid resistance if the barrier effect was an important component of the ITN mode of action. However, evidence of the effectiveness of UTNs to reduce malaria transmission is limited: several observational studies have found UTNs to be protective against malaria [50], but the only community-randomized controlled trial of UTNs compared to no bed nets did not find an epidemiologic benefit, although there was a reduction in blood feeding [51,52]. In this study, there was no significant difference in the incidence of malaria between children using UTNs compared to children not using bed nets, but because UTNs were present in the study community prior to the start of the study, the integrity of their barrier may have been reduced through acquisition of holes.

There are other data to suggest that ITNs provide more than just a physical barrier to malaria transmission in areas with resistant mosquitoes. A recent meta-analysis of the entomologic impacts of pyrethroid resistance found that ITNs were significantly more effective at reducing blood feeding, inducing exophily and killing mosquitoes than UTNs despite pyrethroid resistance [20]. This review found that significant heterogeneity in methods and results across the studies evaluated made it impossible to determine whether ITN effectiveness was affected by the level of insecticide resistance, but in conjunction with the results of this study, it appears reasonable to conclude that the insecticide on ITNs continues to exert a beneficial effect even when a significant degree of pyrethroid resistance is present [20]. Whether the beneficial effect could have been larger in the absence of pyrethroid resistance is not clear.

This is the first study to show that ITNs continue to prevent malaria infections in an area where pyrethroid-resistant *An. funestus* is the dominant vector. As the clearest example to date of operational failure of insecticide-based vector control was in South Africa after *An. funestus* became metabolically resistant to the pyrethroids used in the IRS programme in the late 1990s [10], the results from this study hint at potential differences in the impact of insecticide resistance on ITNs and IRS. As vector control interventions, ITNs and IRS both deter mosquitoes from entering houses, induce exophily and kill mosquitoes that come into contact with the insecticide. However, ITNs also act as baited traps, luring mosquitoes to the insecticide with vapour trails of human body gasses [53], and there are differences between ITNs and IRS in terms of the concentration of insecticide used and the duration of the insecticide that might affect results. It is conceivable that these differences could mitigate the impact of insecticide resistance on ITNs but not IRS. Alternatively, it is possible that the failure of IRS in South Africa was not only due to insecticide resistance: increases in malaria parasite resistance to the first-line anti-malarial treatment (sulphadoxine-pyrimethamine) occurred around the same time and may have played a role in the resurgence of cases [54]. Unfortunately, the available data are not sufficient to clarify the situation.

A weakness in all studies of insecticide resistance and malaria transmission is the actual measurement of resistance. WHO recommends measurement of phenotypic resistance through standardized WHO tube assays or the CDC bottle assay [32]. Both tests measure mosquito mortality after a period of exposure to insecticide. It is recommended to use recently emerged, non-blood fed, adult female mosquitoes to standardize comparisons over time and between sites. However, results from these laboratory assays may not be generalizable to older mosquitoes with infective sporozoites. As they age, mosquitoes may lose the oxidase or esterase enzymes responsible for detoxifying insecticides and become more susceptible to insecticides than younger mosquitoes [55]. As a result, phenotypic assays of recently emerged mosquitoes may overestimate the degree of resistance in the epidemiologic important vectors. However, a more recent study suggests that blood meals cause oxidative stress in resistant mosquitoes that may maintain resistance within a mosquito throughout its life [56]. Due to the low vector density in the area, it was not possible to compare phenotypic resistance assays in wild-caught and recently emerged mosquitoes contemporaneously to determine whether results differed, but this is an area for future exploration.

Aside from the issue of whether existing assays accurately measure the resistance of the mosquitoes responsible for transmitting malaria, it is not clear whether there is a threshold at which resistance may begin to have an epidemiologic impact. Strode and colleagues classified resistance levels into low, medium and high based on phenotypic assays and prevalence of *kdr*; although this somewhat arbitrary classification system was used in this analysis, it is not based on any epidemiologic or entomologic outcomes [20]. It is conceivable that the level of resistance measured in this study is not severe enough to show important epidemiologic effects. Given that resistance levels could increase even more, there will continue to be a need to monitor the impact of resistance on epidemiologic outcomes until and unless, new insecticides or technologies reduce the dependence of ITNs on pyrethroids.

This study had several important limitations. Resistance among mosquito vectors had already occurred in this area so no comparison with ITNs under conditions of full vector susceptibility in this population could be made. ITN use in this population was significantly higher than anticipated, decreasing the precision of the protective effectiveness estimate, and potentially limiting the generalizability of the study results to other areas. The ITNs used in this study were new at the start, and it is possible that effectiveness will decline as bed nets degrade physically. To examine this possibility, the study was extended by an additional nine months; results are being analysed and will be published soon. Use of RDTs for malaria diagnosis during the monthly and sick visits, rather than the gold standard of microscopy, was necessary given the extremely high number of encounters with study participants where malaria infection was measured; but RDTs may pick up treated infections that still have persistent antigen. This problem was assessed by varying the interval for determining whether consecutive positive RDT results were independent, and did not find any difference in interpretation of results.

This study had several strengths, including that it was designed prospectively to answer the question of ITN effectiveness in an area with significant pyrethroid resistance, rather than conceived as a secondary data analysis. A fixed cohort with a high follow-up rate was observed, and ITN use was evaluated frequently over the course of the study with two surveys to examine the reliability of caregiver report of ITN use. Both active and passive surveillance were combined to try to capture all malaria infections, both symptomatic and asymptomatic, and all infections were parasitologically confirmed. Potential confounders were carefully measured and included in the final model, including estimates of malaria exposure and measures of ITN density that may cause a community effect. A recent review lamented the lack of concomitant measures of insecticide resistance in other studies of ITNs and pyrethroid resistance [20]; this study collected information before and during the study to ensure that insecticide resistance measurements were characteristic of the vector species transmitting malaria in the same time and place as the study participants, with the caveat that only recently emerged mosquitoes were evaluated. Additionally, the mechanism of insecticide resistance underpinning the phenotypic results was identified.

## Conclusion

ITNs continue to protect children from malaria in an area of Malawi with very high reported ITN use, moderate insecticide resistance and high rates of malaria transmission, annually preventing nine cases of malaria for every ten children protected. Based on these findings, national malaria control programmes should continue to procure and distribute ITNs for areas with moderate insecticide resistance. However, as the measures of insecticide resistance used in this investigation are imperfect, and distribution and intensity of insecticide resistance could still increase, these findings do not suggest complacency with the *status quo*. Periodic monitoring of the effectiveness of ITNs in areas of moderate to high pyrethroid resistance should continue, while research on new insecticides and new technologies should be pursued urgently to eliminate the pyrethroid Achilles’ heel of ITNs.

## Abbreviations

CI: Confidence interval
Hb: Haemoglobin
HRP-2: Histidine-rich protein 2
IDW: Inverse-distance weighted
iPPY: Incidence per person-year
IQR: Interquartile range
ITN: Insecticide-treated bed net
*kdr*: Knockdown resistance
RDT: Rapid diagnostic test
PBO: Piperonyl butoxide
PCR: Polymerase chain reaction
PE: Protective effectiveness
PSC: Pyrethroid spray capture
PYAR: Person-years at risk, RR, rate ratio
UTN: Untreated bed net
WHO: World Health Organization
